# Supplementary material for: Qualitative community stability determines parasite establishment and richness in estuarine marshes
Source: PeerJ. 2013 Jun 18;1:e92. doi: 10.7717/peerj.92 (PMC3691787; doi:10.7717/peerj.92)
Supplement: Supplemental Information — Methods describing community stability analyses. [file peerj-01-92-s001.docx]

**SUPPLEMENTARY METHODS**

*(a) Community stability analyses*

Food web stability was assessed using the framework provided by (May 1972; 1973) and extended by (Allesina & Pascual 2008). It is based on the concept of food web matrices and the dynamics of species densities in the network, determined by the equations:

$\frac{dX_{i}}{dt}=F_{i}(X_{1}(t),X_{2}(t),X_{3}(t),...,X_{n}(t))$. (1)

where *X_i_* is the abundance of species *i* and *n* is the total number of species. The steady state of the system, in which all growth rates are zero, occurs when:

$0=F_{i}(X_{1}^{*}(t),X_{2}^{*}(t),X_{3}^{*},...,X_{n}^{*}(t))$. (2)

Expanding about this equilibrium, for each population:

$x_{i}(t)=X_{i}(t)-X_{i}^{*}$. (3)

where *x_i_* measures a small perturbation to the *i*th population and * denotes the steady state. The dynamics and stability in the neighborhood of the steady-state point may be determined by expanding (1) in a Taylor series about the steady state, discarding all terms that are of second or higher order in the population perturbations *x*. A linearized approximation may be obtained, in matrix notation:

$\frac{d\text{ }\mathbf{x}(t)}{dt}=A\text{ }\mathbf{x}(t).$ (4)

where **x**(*t*) is an *n* × 1 column vector of the deviations from the steady state. May (1973) demonstrates that the eigenvalues for *A* reveal the stability properties of the system. Specifically, if matrix *A* is stable, its eigenvalues will all have negative real parts.

To compute the eigenvalues for our sites we generated community matrices from adjacency matrices that describe the trophic interactions from the marshes in which we work (Anderson 2009; Anderson & Sukhdeo 2011). In order to convert these matrices into the matrices described by May (1973) we followed the methods of Allesina & Pascual (2008): if *a_ij_* > 0 then *a_ji_* < 0 for each *i* $\neq$ *j*, in doing so creating an antisymmetric matrix. The diagonal coefficients of the matrix were set to -1 (self-regulation). Coefficient strengths were assigned by extracting values from a standard normal distribution ($\mu$ = 0, $\sigma^{2}$ = 1), taking the absolute value, and multiplying the antisymmetric community matrices with the randomly determined interaction coefficient. For each of the matrices we calculated the percentage of eigenvalues with a negative real parts; further, we randomized the assignment of coefficients 100,000 times in order to determine whether the eigenvalues we obtained were due to combinations of coefficients or a property of trophic structure of the food webs.

**REFERENCES**

Allesina, S. & Pascual, M. (2008) Network structure, predator-prey modules, and stability in large food webs. *Theoretical Ecology*, **1**, 55–64.

Anderson, T.K. (2009) *Food Web Networks and Parasite Diversity*. Rutgers University.

Anderson, T.K. & Sukhdeo, M.V.K. (2011) Host centrality in food web networks determines parasite diversity. *PLoS ONE*, **6**, e26798.

May, R. (1972) Will a large complex system be stable? *Nature*, **238**, 413–414.

May, R. (1973) *Stability and Complexity in Model Ecosystems*. Princeton University Press, Princeton.
